# 

**Published:** 1908-02

**Authors:** 


### CONTENTS.

ORIGINAL COMMUNICATIONS—
Education and the Social Evil. By Alex. W. Stirling, M. D., C. M., D. P. H., Atlanta, Ga...637
The Perfect Mechanical Reduction of Fractures and Method of Absolute Immobilization. By J. H. Downey, Gainesville, Ga. .. .. .. .. .. .. .. .. .. .. .. .. .. .. .. ..645
The Importance of Testing the Ocular Muscles. By R. B. Ridley, Jr., M. D., Clinical Professor of Eye, Ear, Nose and Throat, Atlanta School of Medicine. .. .. .. .. .. ..656
Albuminuria. By Edgar Ballenger, M. D., Atlanta, Ga., Lecturer on Genito-urinary Diseases, Atlanta School of Medicine. .. .. .. .. .. .. .. .. .. .. .. .. .. ..659
CORRESPONDENCE—
EDITORIAL NOTES AND COMMENTS—
The Atlanta Medical Library Association. .. .. .. .. .. .. .. .. .. .. .. ......666
Medical Societies and Charlatans. .. .. .. .. .. .. .. .. .. .. .. .. .. ..667
The Death of Nicholas Senn. .. .. .. .. .. .. .. .. .. .. .. .. .. ....669
NEWS AND NOTES. .. .. .. .. .. .. .. .. .. .. .. .. .. .. .. .. ..670
BOOK REVIEWS. .. .. .. .. .. .. .. .. .. .. .. .. .. .. .. .. .... 672
SELECTIONS AND ABSTRACTS. .. .. .. .. .. .. .. .. .. .. .. .. ..675 to 694

